# Effect of Non-Coal Heating and Traditional Heating on Indoor Environment of Rural Houses in Tianjin

**DOI:** 10.3390/ijerph16010077

**Published:** 2018-12-29

**Authors:** Liansheng Liu, Hua Yang, Runze Duan, Minghai Liu, Ruifang Zhang, Yiji Ding, Hongzhen Sun

**Affiliations:** 1 School of Energy and Environmental Engineering, Hebei University of Technology, Tianjin 300401, China; liuliansheng@hebut.edu.cn (L.L.); dhbdrz@163.com (R.D.); r13373251502@163.com (R.Z.); dingyijitd@163.com (Y.D.); sunhz97@163.com (H.S.); 2 China Railway Construction Group Co., Ltd., Beijing 100043, China; 17720164375@163.com

**Keywords:** indoor air quality, non-coal heating, coal-fired heating, rural house, indoor heat and humidity environment, pollution emission

## Abstract

In order to understand the effect of the non-coal heating and the traditional coal-fired heating on the indoor environment of the rural houses, the humidity environment and indoor air quality in several households were investigated during the heating period in Beichen District and Wuqing District of Tianjin, China. The results showed that the indoor average temperature for the heating by the electricity and the natural gas was higher than that by the traditional coal fire. The indoor relative humidity for the heating by the electricity and the natural gas was lower than that by the traditional coal fire. The indoor air quality (IAQ) for the heating by the electricity and the natural gas was better than that by the traditional coal fire. For traditional coal-fire heating households, the indoor pollutant emission (CO emission) by using the clean coal was lower than that by using the raw coal. The indoor ventilation rate which was an important parameter for the indoor air quality was generally poor in winter. The total volatile organic compounds (TVOC) emission in the indoors of the coal-fired heating households was generally higher than that of the non-coaled heating households.

## 1. Introduction

Rural population was about 605 million in China, about 44% of the total population. In winter, the farmers spent 80% of their time indoors, the indoor air quality (IAQ) was very important for the health of the farmer. It was necessary to test and analyze the indoor and outdoor air quality [1]. At present, many researchers had studied the indoor air quality of the northern rural houses in China. Wang et al. [2] investigated the indoor thermal environment of the farmhouses in summer and winter. The results found that the indoor air quality was bad, especially in winter. Padilla et al. [3] evaluated the indoor air quality of the farmhouses in severe cold regions in winter. The pollutants in the farmhouse were particles, NO_x_ and CO. Sun et al. [4] investigated the IAQ in Tianjin residential buildings, China. The conclusions were that outdoor particles and indoor activities both had impact on indoor particulate matter. Wolkoff [5] reviewed about indoor air humidity, air quality and health. The results showed that elevation of the indoor air humidity may negatively impact the IAQ. Some researchers [6,7,8,9,10,11,12,13,14] found more resuspension of large particle and less for smaller particles at low relative humidity.

All the literatures mentioned above mainly focused on the IAQ. The IAQ was mainly affected by the ventilation rate. The indoor ventilation was investigated by many researchers. Lei et al. [15] investigated the effect of the natural ventilation in student dormitories on indoor air quality and thermal comfort in Beijing, China. The results found the IAQ cannot meet the comfortableness only by opening the windows.

In recent years, the smog weather in northern China has appeared frequently during the heating periods. In order to cope with the smog pollution and improve the air quality, the government began to implement non-coal heating policy in 2017, for example coal to natural gas, coal to electricity and so on. It was necessary to study the effect of the policy on the IAQ. Therefore, the objective of this paper was to test and compare the IAQ of the non-coal heating and the traditional coal-fired heating in Tianjin. It is hoped that the present work can provide a good reference for the farmhouse heating in this region.

## 2. Method

In order to understand the effect of the non-coal heating and the traditional coal-fired heating on the IAQ in rural regions of Tianjin, this paper selects typical farmhouses with different heating model to test their IAQ. To ensure that the selected samples are representative, four hundred questionnaires were conducted on rural residents in Wuqing (200) and Beichen (200) Districts in September 2017. Wuqing district have implemented “no coalification” transformation in Tianjin and Beichen district have not been reformed. The questionnaire responses mainly include family information, housing situation and heating information. The general situation of Tianjin rural areas is summarized as follows:

1. The annual income of every family is basically below 12,000 $ per year and the number of permanent residents of every family is mostly 2 to 3 and 33.2% of the families have old people or children.
2. Most of the farmhouses which were built in the 1990s are independent courtyards, north-south orientation. The exterior wall materials are solid bricks, the wall thickness is mostly 370 mm and some 240 mm. The windows are mostly single-layer wooden or aluminum alloy materials and no thermal insulation equipment.
3. The main heating modes are burning coal, electricity heating and burning natural gas.
4. Temperature (t) and relative humidity (RH) were obtained by RC-4HA/C Temperature and Humidity Recorder which made in China with the accuracy of ±0.5 °C and ±3% RH, respectively. The indoor particles PM_2.5_ and PM_10_ are measured by CEL-712 (IDEAL INDUSTRIES, Sycamore, USA) Microdust Pro which made in England with the accuracy of 0.001mg/m^3^. The indoor CO and CO_2_ emission are obtained by TsI7545 (TSI, Minnesota, USA) which made in America with the accuracy of 3%. The indoor TVOC emission is measured by SIGNAL Model 3010 (SIGNAL, London, England) which made in England with the accuracy of 1%.

The objective of the paper is to analyze and compare the indoor temperature, relative humidity and IAQ parameters for the non-coal heating and the traditional coal-fired heating farmhouses in Tianjin. Considering the building structural characteristics, the resident economic level, the living habits and the heating status quo in rural areas of Tianjin, the seven typical households were selected as research objects.

### 2.1. Households Information

The typical seven households were chosen from 400 questionnaires. The seven households are all independent courtyards, north-south orientation. The houses were built by using solid brick in 1990s. The thicknesses of the exterior walls are 370 mm and the walls were not furnished with thermal insulation equipment. The doors and windows are single-layer wooden material. The height of the house is between 3.0 and 4.0 m and the heated area is between 40 m^2^ and 80 m^2^. These households include three households which adopt the traditional coal fire for heating in winter in Beichen District, two households which adopt electric heating in winter in Wuqing District and the others which adopt natural gas for heating in Wuqing District. The detail information of the households is shown in Table 1. The households from No.1 to No.3 in the Table 1 are traditional coal-fired heating. The household No.4 is a combination of traditional coal-fired heating and natural gas heating. The household No.5 is a natural gas heating. The households No.6,7 are electric heating.

### 2.2. Test Methods for IAQ Evaluation

The average room area of the general farmhouses is about 15~20 m^2^. According to the selection principle of the sampling points in the test indoor hot and humid environment, the measuring point can be set in the center of the room where the coal stove is placed and the adjacent room. The height of the measuring point is 1.5 m from the ground, which is the height of the human breathing zone. The main sources of pollutants in the farmhouse and the harm to the human are considered. The test parameters include temperature (t), relative humidity (RH), air flow rate (v), particulate matter, respirable particulate matter, carbon monoxide (CO), carbon dioxide and total volatile organic compounds (TVOC) in this paper.

The test period was from November 2017 to mid-March 2018 for a total of 158 days. The temperature and humidity were continuously monitored during the test process and the remaining parameters were tested every 7 to 10 days. The duration of each measurement is maintained at about 30 to 60 min. In order to avoid the influence of some factors, such as firing, cooking and cleaning and so on, the test process is carried out in the afternoon.

## 3. Result and Analysis

In order to understand the basic situation of indoor heating, humidity environment and air quality in the different conditions of the non-coal heating and the traditional coal-fired heating, the test parameters of the indoor environment were collected, as shown in Table 2.

### 3.1. Indoor Heat and Humidity Environment

The indoor temperature and humidity of the farmhouse is measured by the RC-4HA/C temperature (HUAHENG, Chengdou, China) which made in China and humidity recorder which the temperature with the accuracy is 0.5 °C of the full scale and the relative humidity with the accuracy 3% of the full scale. The measurement results are recorded every fifteen minutes. The indoor temperature and relative humidity standards in China are 16–24 °C and 30%–60%, respectively. The indoor wind speed is obtained by the testo425 thermal anemometer (HUAHENG, Chengdou, China) which made in Germany with the accuracy 5% of the full scale. The standard of the indoor air flow velocity in China is 0.2 m/s.

#### 3.1.1. Temperature

**Figure 1:**
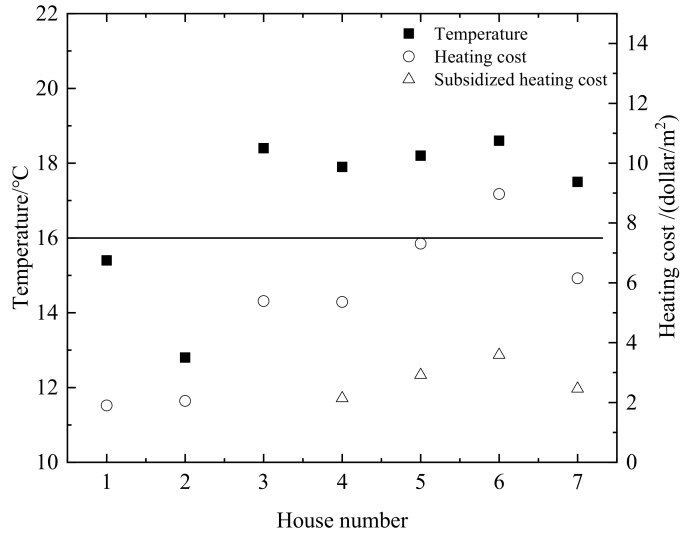
Indoor average temperature and annual heating costs during heating period.

During the heating period, the annual heating costs and the indoor average temperature in different households are shown in Figure 1. The indoor temperature of the non-coal heating households is generally higher than that of the traditional coal-fired heating used by farmers. However, the annual heating costs of the non-coal heating households also are higher than that the coal-fired heating households. The government will subsidize 60% of the non-coal heating costs in China. The annual heating costs of the non-coal heating households will be greatly reduced, as shown in Figure 1. The indoor temperature of the No.3 household should ideally be higher than other coal-fired heating households, because there is a child in this household. So, the annual heating costs are higher than others.

Comparing the indoor temperature and the annual heating costs of No.1 and No.2 coal-fired heating households, it is found that the annual heating costs of the two households are slightly different. The indoor temperature of No.2 household is significantly lower than that of No.1 household. The reason is that the heating equipment of the No.1 household is the radiator equipment and the heated brick bed. The heating equipment of the No.2 household is the coal stove and the heated brick bed. The radiator equipment will greatly affect the indoor temperature. The No.4 and No.5 are “coal to natural gas” households. Comparing the indoor temperature and the annual heating costs of the two households, it is found that the indoor temperature of the two households is slightly different but the annual heating costs of the No.5 household is significantly higher than the No.4 household. Though the housing structures of No.4 and No.5 households are similar, the No.5 household is only heated by the radiator equipment and the No.4 household uses the radiator equipment, the heated brick bed and the coal stove. Therefore, for the “no coalification” households, the auxiliary heating sources such as heated brick bed can save the heating costs, meanwhile, the indoor temperature can satisfy the residents’ requirement for warmth.

**Figure 2:**
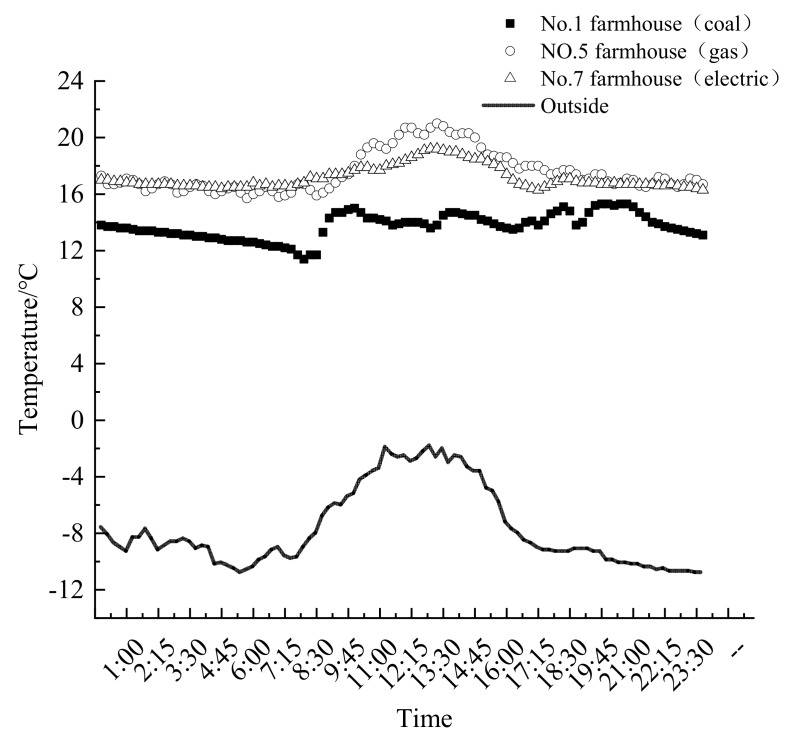
Indoor temperature changes of the three households.

During the heating period, the temperature tendencies are same for the different households. The indoor and outdoor temperature changes for the different households in the day and night are chosen, as shown in Figure 2. The heating methods are coal-fire heating (No.1), electricity heating (No.5) and natural gas heating (No.7), respectively. These households have the radiator equipment. The indoor temperature of the electricity heating and natural gas heating is higher and more stable than that of the coal-fire heating under the same outdoor temperature condition. The reason is that the non-coal heating is continuous at day and night. The temperature will not significantly decline when the residents fall into sleep at night. For the traditional coal-fire heating, the temperature is high from 9:00 am to 10:00 pm, which corresponds to the residents heating during the day and the indoor temperature is maintained at 14 °C. However, the indoor temperature would decline because the residents stopped using the coal fire between 10:00 pm and 9:00 am the next day, decline to 11 °C. According to the above analysis, the indoor temperature of the non-coal heating households is generally higher than that of the traditional coal-fired heating households and keeps proper sleep temperature at night. However, the indoor average temperature is low for the coal-fire heating. The thermal comfort of the residents will be greatly reduced.

#### 3.1.2. Relative Humidity

**Figure 3:**
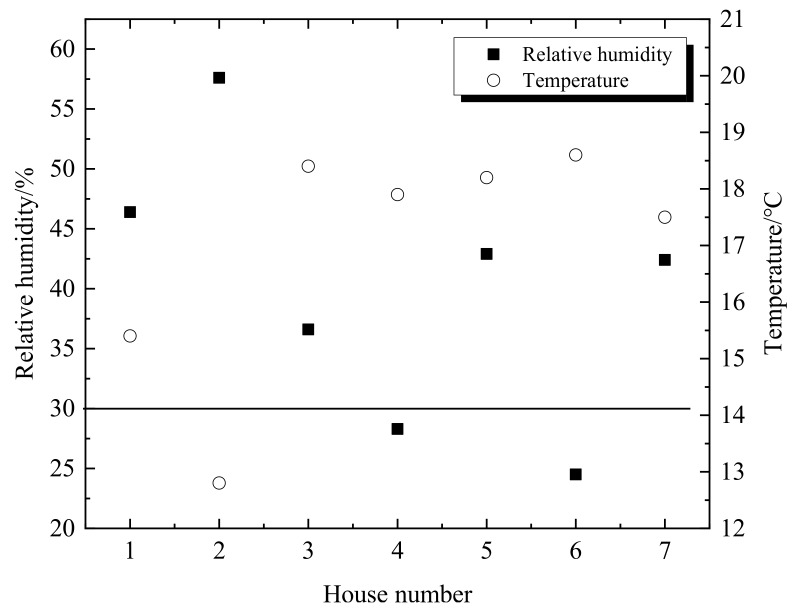
Indoor temperature and relative humidity during heating period.

The relative humidities of the different households varied greatly from 25% to 58%, as shown in Table 2. The relative humidity of the No.4 and No.6 households which are non-coal heating is lower than the national standard (30%) in China. The indoor temperature and the air moisture content directly affect the relative humidity. The relative humidity values of the different households are shown in Figure 3. The indoor relative humidity of the non-coal heating is lower than that of the traditional heating. The relation of the temperature and the relative humidity is inversely proportional. However, the relation of the air moisture content and the relative humidity is proportional. The temperatures of the No.3 and No.6 are somewhat similar but the indoor humidity of the No.3 is higher than that of the No.6. The reason is that the stove was used to heat the hot water for residents living in No.3 households. The water evaporation can increase the indoor air moisture content. There are some potted plants in the No.5 and No.7 households. The evaporation of water in the soil and plant leaves increases the indoor air moisture content. So, the relative humidity values of the No.5 and No.7 households were relatively high. The No.4 and No.6 households have no humidification and the relative humidity is lower than the national standard (30%) in China. In winter, the outdoor air in the northern of China is relatively dry, the indoor temperature is relatively high. The indoor relative humidity needs to be increased in order to improve the comfort of residents.

### 3.2. Indoor PM_2.5_ and PM_10_ Emission

The indoor particles PM_2.5_ and PM_10_ are measured by CEL-712 Microdust Pro which made in England with the accuracy of 0.001 mg/m^3^. The national standards in China of PM_2.5_ and PM_10_ are 0.075 mg/m^3^ and 0.15 mg/m^3^, respectively. The results of the PM_2.5_ and PM_10_ are measured, as shown in Table 2. The indoor particles PM_2.5_ and PM_10_ exceed the national standards in China except No.5 and No.7. The reason is that the No.5 and No.7 households adopted non-coal heating in winter. The pollution emission was low through fossil fuel combustion. There were some plants in No.5 and No.7 and the plants can adsorb some particles [16]. So, there are few particles in the No.5 and No.7 households.

**Figure 4:**
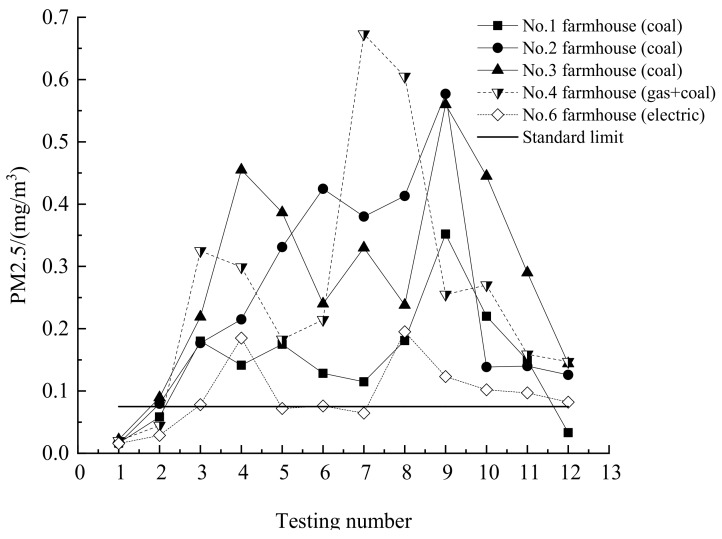
Changes in PM_2.5_ content in various households.

The coal-fire heating was adopted from No.1 household to No.4 household in winter. The different coal types lead to the different pollution. The plumbing air conditioner was used for heating in the No.6 household. There are a lot of dusts in farmhouse. The heating method will promote the air flow. The particles will increase in the room. The changes tendencies of the PM_2.5_ are same as the PM_10_. So, the measurement results of the PM_2.5_ are analyzed herein. The particles PM_2.5_ are measured in different period, as shown in Figure 4. The solid line is the national standards in China. The first test is no heating period. There are few particles PM_2.5_ in the room. The second test is the early period of the heating. The particles PM_2.5_ sharply increase for the coal-fire heating in the heating period. The combustion process of the coal made the increase of particles PM_2.5_. The particles PM_2.5_ greatly exceed the national standards in China. The particles of the No.6 household are relatively small. The heating for the No.6 household adopts electricity. The 11th and 12th tests are the end period of the heating. The indoor particles PM_2.5_ will greatly decline. The results are that the indoor particles PM_2.5_ mainly produced by the coal combustion. The indoor particles PM_2.5_ in the non-coal heating are relatively low.

### 3.3. Indoor CO and CO_2_ Emission

The indoor CO and CO_2_ emission are measured by TsI7545 (TSI, Minnesota, USA) which made in America with the accuracy of 3%. The indoor CO and CO_2_ emission standards in China are 10 mg/m^3^ and 1964.3 mg/m^3^, respectively.

**Figure 5:**
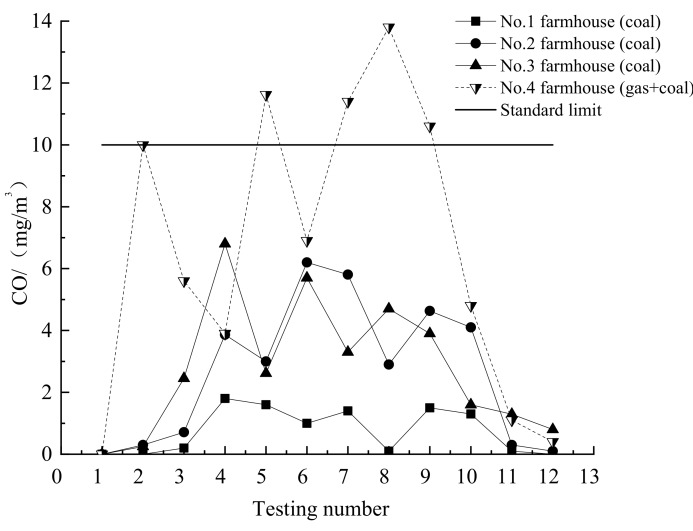
Changes in CO content in coal-fired farmhouses.

The results of the CO and CO_2_ emissions are measured, as shown in Table 2. Most of the values for CO emission are lower than the national standard in China. The reason is that the airtightness of rural houses is generally poor. The indoor CO emission of the coal-fire heating is higher than that of the non-coal heating. The CO emission from the No.1 is shown in Figure 5. The CO emission is lower than others. The reason is that household heating adopts the clean coal and the CO emission relatively small. The CO emission of the No.4 household is higher than others, sometimes exceed the national standard in China. The reason is that the No.4 household has no smoke exhaust pipes. The CO is directly emitted into the room.

CO_2_ is an indicator of the indoor ventilation. The CO_2_ emission is good except the No.3 household, as shown in Table 2 There is a child in the No.3 household. In order to keep the indoor temperature, there is almost no ventilation in winter. The others have ventilation due to the living habits. The outdoor CO_2_ emission below 650 mg/m^3^ is much lower than the indoor CO_2_ emission. This indicates the indoor ventilation is generally poor in winter.

### 3.4. Indoor TVOC Emission

The indoor TVOC emission is measured by SIGNAL Model 3010 (SIGNAL, London, UK) which made in England with the accuracy of 1%. The indoor TVOC of the national standards in China is 0.60 mg/m^3^.

**Figure 6:**
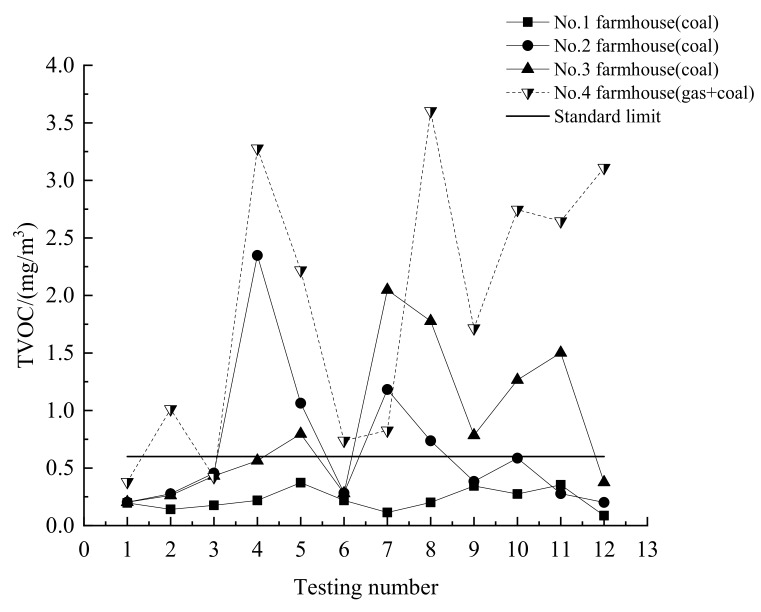
Content changes of TVOC in coal-fired farmhouses.

The TVOC which contained halogenated hydrocarbon and aldehyde can be harmful to the human body when indoor emissions are too high. The TVOC emission in the indoors of the coal-fired heating households is generally higher than that of the non-coaled heating households and there is a phenomenon of exceeding the standard, as shown in Table 2. The indoor TVOC is produced by decoration and fuel burning. In these tests, the farmhouses have not been renovated in the past 5 years. So, the indoor TVOC emission is mainly produced by the coal-fired heating. The indoor TVOC emission from No.1 to No.4 is measured, as shown in Figure 6. The indoor TVOC emission exceeds the national standards in China except the No.1 household. The indoor TVOC emission of the No.4 household is most serious. The reason is that the heating of the No.1 household adopts the clean coal whereas the paint bucket was as a stove for heating in No.4 household which had no smoke exhaust window. The paint will release the volatile vapors during the heating period due to a significant increase in indoor TVOC emission.

## 4. Conclusions

In this paper, the indoor air quality of 7 typical households in Beichen District and Wuqing District of Tianjin were compared on the basis of different home heating methods. In order to reduce or eliminate the presence of polluting compounds, eco-sustainable systems and plant techniques at low emissions and reduced energy consumption could be used. The effect of the coal-fire heating and the non-coal heating on the indoor air quality is analyzed. Some conclusions are obtained as follows,

(1) During the heating period, the average indoor temperature of the traditional coal-fired heating is generally lower than 16 °C. The indoor temperature even drops to a lower level at night. The indoor temperature of the non-coal heating households is generally higher than that of the traditional coal-fired heating, which is basically above 17 °C and the heating generally works at day and night, which can ensure the proper temperature.
(2) For non-coal heating households, the indoor relative humidity may be less than 30% and the indoor environment is dry without taking humidification measures. It is recommended that these households take appropriate measures to increase the indoor relative humidity.
(3) The indoor pollutant emission of the non-coal heating households is generally lower than that of the traditional coal-fired heating households. The change of the heating energy can effectively improve the indoor air quality.
(4) For the traditional coal-fired heating households, the indoor pollutant emission of the households using clean coal heating is significantly lower than that of the households using non-clean coal heating. The use of the clean coal can effectively reduce indoor pollutant emission.

## Figures and Tables

**Table 1:** Households information statistics in rural region of Tianjin.

Households Number	Family Member	Heating Information	Characteristics
1	4 permanent residents, aged between 18 and 65 years old	Heating energy is a new type of clean coal, heating method is radiator + heated brick bed	The heating energy is a new type of clean coal
2	2 permanent residents, aged between 40 and 65 years old	Heating energy is traditional raw coal and the heating method is coal stove + heated brick bed	No radiator installed in doors
3	3 permanent residents, a two-year old child and the remaining members between 40 and 65 years old.	Heating energy is traditional raw coal, heating method is radiator + heated brick bed	The heating energy is raw coal and the stove heats the hot water for living
4	2 permanent residents, aged between 40 and 65 years old	“Coal to natural gas” users, Heating energy is natural gas combined with traditional raw coal, Heating method is radiator + heated brick bed	The heating energy is natural gas combined with traditional raw coal, no smoke exhaust windows. The stove is produced by paint bucket.
5	2 permanent residents, aged between 40 and 65 years old	“Coal to natural gas” users, heating method is the radiator	The heating energy is natural gas and there are some potted plants in the room.
6	3 permanent residents, aged between 40 and 65 years old	“Coal to electricity” users, heating equipment is the plumbing air conditioner	The heating energy is electricity
7	2 permanent residents, aged between 40 and 65 years old	“Coal to electricity” users, heating method is radiator	Heating method is radiator and there are some potted plants in the room.

**Table 2:** Test results of various IAQ parameters.

Test Parameters	Test Farm Number						
	1	2	3	4	5	6	7
t/°C	15.4	12.8	18.4	17.9	18.2	18.6	17.5
RH/%	46.4	57.6	36.6	28.3	42.9	26.5	42.4
V/(m/s)	0.07	0.06	0.04	0.07	0.07	0.06	0.05
PM_2.5_/(mg/m³)	0.143	0.287	0.282	0.291	0.045	0.117	0.047
PM_10_/(mg/m³)	0.224	0.341	0.317	0.464	0.069	0.152	0.076
CO/(mg/m³)	0.7	3.0	3.1	6.8	0	0	0.2
CO_2_/(mg/m³)	1458	1827	2550	1883	1770	1433	1658
TVOC/(mg/m³)	0.4	0.7	1.1	1.9	0.1	0.2	0.2
